# The Quest for EEG Power Band Correlation with ICA Derived fMRI Resting State Networks

**DOI:** 10.3389/fnhum.2013.00315

**Published:** 2013-06-25

**Authors:** Matthias Christoph Meyer, Ronald Johannes Janssen, Erik Sophius Bartus Van Oort, Christian F. Beckmann, Markus Barth

**Affiliations:** ^1^Radboud University Nijmegen, Donders Institute for Brain, Cognition and Behaviour, Nijmegen, Netherlands; ^2^MIRA Institute for Biomedical Technology and Technical Medicine, University of Twente, Twente, Netherlands; ^3^Erwin L. Hahn Institute for Magnetic Resonance Imaging, University Duisburg-Essen, Essen, Netherlands

**Keywords:** combined EEG-fMRI, resting state, source modeling, RSN, ICA, ECP, IHM

## Abstract

The neuronal underpinnings of blood oxygen level dependent (BOLD) functional magnetic resonance imaging (fMRI) resting state networks (RSNs) are still unclear. To investigate the underlying mechanisms, specifically the relation to the electrophysiological signal, we used simultaneous recordings of electroencephalography (EEG) and fMRI during eyes open resting state (RS). Earlier studies using the EEG signal as independent variable show inconclusive results, possibly due to variability in the temporal correlations between RSNs and power in the low EEG frequency bands, as recently reported (Goncalves et al., 2006, 2008; Meyer et al., 2013). In this study we use three different methods including one that uses RSN timelines as independent variable to explore the temporal relationship of RSNs and EEG frequency power in eyes open RS in detail. The results of these three distinct analysis approaches support the hypothesis that the correlation between low EEG frequency power and BOLD RSNs is instable over time, at least in eyes open RS.

## Introduction

Blood oxygen level dependent (BOLD) functional magnetic resonance imaging (fMRI) resting state networks (RSNs) have increasingly generated interest in the neuroscientific community, but the neuronal underpinnings remain unclear so far. Early studies, which examine correlations between the electroencephalography (EEG) theta, alpha, or beta band power and BOLD signal fluctuations using EEG derived regressors (Goldman et al., 2002; Laufs et al., 2003a,b, 2006; Moosmann et al., 2003; Feige et al., 2005; Goncalves et al., 2006; Scheeringa et al., 2008), report rather mixed and inconclusive BOLD correlation maps. The discovery and further analysis of RSNs (Biswal et al., 1995; Lowe et al., 2000; Cordes et al., 2001; Greicius et al., 2003; Fox et al., 2005; Damoiseaux et al., 2006; De Luca et al., 2006; Smith et al., 2009), together with the above mentioned early combined EEG-fMRI studies gave rise to the assumption that several frequency bands might be involved in distinct functional networks (Laufs et al., 2006; Mantini et al., 2007). The replication of this finding on subject level would fundamentally improve our understanding of the link with electrophysiology.

Simultaneous recordings of EEG and fMRI during resting state (RS), enables the investigation of the electrophysiological correlates of BOLD RSNs. Using simultaneous recordings, Mantini et al. (2007) reported a specific EEG frequency band power signature for RSNs on group level in eyes closed RS. However, further studies show large inter-subject variations of distinct brain areas correlated with EEG alpha band power (Goncalves et al., 2006, 2008) in RS. In a recent study by Meyer et al. (2013) electrophysiological correlation patterns (ECPs) between RSN BOLD time courses and EEG frequency band power showed large inter-subject and within subject variability. While RSNs by themselves exhibit a high reproducibility of their spatial characteristics across subjects, these studies point to less stable temporal correlations between RSNs seen in BOLD fMRI and EEG frequency band power.

Based on this evidence we hypothesize that the relationship between EEG frequency band power and RSN BOLD time courses is not stable over time. In order to assess this temporal variance in the correlation of the EEG signal and RSNs within a subject in this study, a dataset with a long RS of 34 min was split up into 15 segments and each was analyzed using the following three analysis approaches:

(1) Global frequency power correlation (GFPC) (Meyer et al., 2013) resulting in ECPs an approach that is similar to the one used by Mantini et al. (2007) who found stable correlation patterns on group level.
(2) An extended version of this method, including an anatomically informed analysis (Dale et al., 2000; Ou et al., 2010; Janssen et al., 2012) to separate the EEG based on RSN *Z*-maps within a subject, to obtain source frequency power correlation (SFPC), which should reduce the effect of volume conduction in the EEG.
(3) A channel wise frequency power fit (CFPF) with minimal assumptions, using the BOLD RSN time courses as the independent variable, which further reduces methodological bias.

We then calculated the temporal variance over the 15 segments for each of the three methods to estimate the temporal stability of the correlation between the two modalities.

## Materials and Methods

### Data acquisition and pre-processing

In this study we performed a new analysis of the data sets acquired in Meyer et al. (2013). We briefly summarize the acquisition protocol and the pre-processing steps (for details, see Meyer et al., 2013): 34 min of eyes open RS were recorded from 12 healthy subjects, using combined EEG-fMRI, with approval of the local ethical committee. MR data were acquired on a 3 T Magnetom TIM Trio system (Siemens Healthcare, Erlangen, Germany) using the product 32 channel head coil. Functional data were recorded using a multi echo EPI sequence (Poser et al., 2006) (1030 Vol., TR = 2000 ms, 3.5 mm isotropic voxel size). A T1-weighted structural scan (MPRAGE) at 1 mm isotropic voxel size was also obtained (with EEG cap), to register the functional data to Montreal neurological institute (MNI) space. Five of the subjects (subjects 1, 2, 4, 10, and 11) were invited back to acquire a second T1-weighted structural scan without the EEG cap to enable the head model based analysis.

Simultaneous EEG data were recorded with a 32 channel cap (ANT WaveGuard MRI), using a BrainAmp MR plus amplifier (250 Hz low-pass analog hardware filter, 10 s time constant, 5 kHz sampling rate, 0.5 μV resolution, reference electrode: FCz) and BrainVision Recorder (BrainVision, Gilching, Germany). Two of the subjects were recorded with a 64 channel cap (BrainVision) using two BrainAmp MR plus amplifier; the same 30 channels (10–20 system) were used for all subjects in the analysis. The subjects were asked to relax, keep their eyes open, stay awake, and not think of anything specific. The room was darkened during the scan and an infrared eye tracker was used to confirm that the subject did not fall asleep. All subjects managed to stay awake for the complete duration of the experiment.

Functional magnetic resonance imaging pre-processing was performed using functions from the SPM5 software package (Welcome Department of Imaging Neuroscience, University College London, UK). The five echoes acquired at every time point were combined after SPM5 motion correction (Poser et al., 2006).

Electroencephalography pre-processing: MR related artifacts in the EEG signal were removed using Analyzer 2 (BrainVision). Trigger based average subtraction (Allen et al., 2000), as implemented in Analyzer 2, was applied to correct for gradient artifacts. The data were filtered using a Butterworth zero phase filter, 48 dB/oct, with a low cutoff at 0.8 Hz, to remove slow fluctuations from respiration, and a high cutoff at 50 Hz. Additionally, a notch filter at 50 Hz was used to remove residual mains frequency noise. Cardiac related MR artifacts were removed using the adaptive average subtraction (AAS) method of Analyzer 2 in semiautomatic mode (Allen et al., 1998). Further, eye blink related artifacts were removed using ICA and the EEG data were re-referenced to a common average.

### Analysis

**Figure 1:**
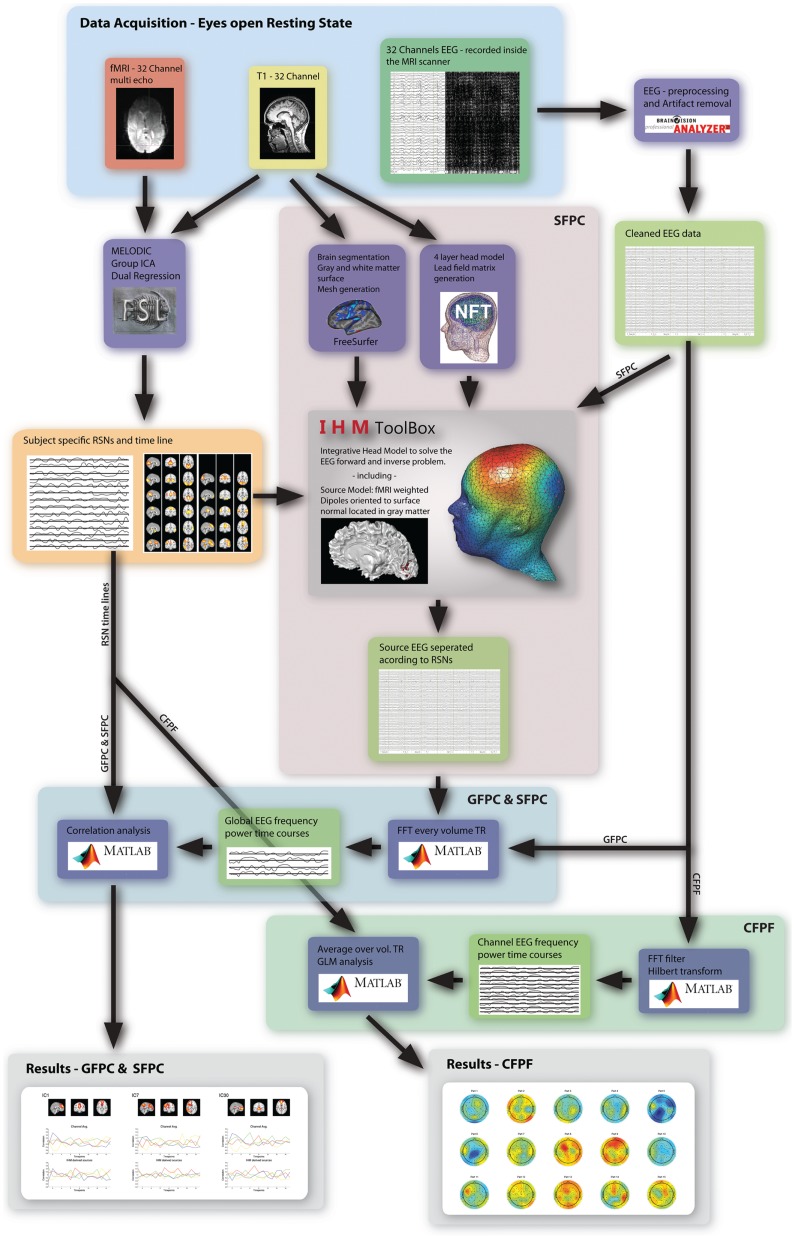
**Overview of the three analysis methods used in this study**. The highlighted regions and arrows are labeled accordingly.

**Figure 2:**
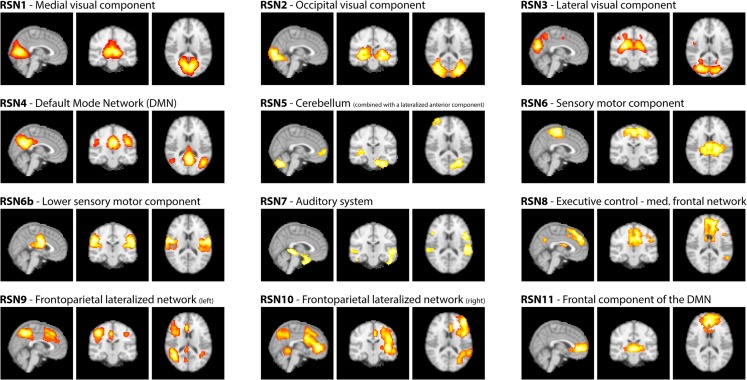
**RSNs on group level as maximum intensity projection on the central slices and their classification according to Smith et al. (2009)**.

As motivated in the introduction, three distinct methods (see Figure 1) were used to infer whether the relationship between EEG frequency band power and RSN BOLD time courses is temporally instable. For all these methods, the preprocessed fMRI data were spatially smoothed by 5 mm and transformed to MNI space using FMRIB’s Software Library’s (FSL) Feat (version 4.1^1^; Smith et al., 2004; Woolrich et al., 2009; Jenkinson et al., 2012). Group independent component analysis (ICA, as implemented in the FSL tool Melodic version 3.1) was performed on the fMRI data to obtain 30 ICs and 12 task related RSNs were selected according to Smith et al. (2009), see Figure 2 for a depiction of the RSNs. A dual regression approach was used to derive subject specific RSN maps and time courses (Filippini et al., 2009). The further analysis is described for each method separately below.

### Global frequency power correlation

**Figure 3:**
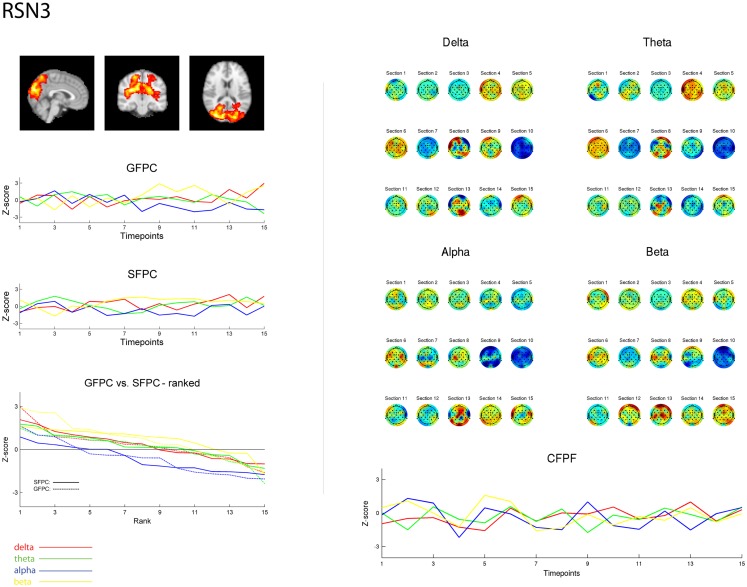
**Exemplary representation of data for the three methods used, depicting the results for RSN3 (lateral visual component) of subject 1**. On the upper left hand side the subject specific map of RSN3 is plotted as maximum intensity projection on the central slices. The two graphs in the middle show the ECP time courses for each frequency band for GSPC and SFPC, illustrating the temporal variance. In the rank graph below the two methods are plotted on top of each other, depicting the similar variance of SFPC (solid line) and GFPC (dot-dashed line); for both methods alpha shows more negative correlation with BOLD in this visual component. On the upper right hand side the temporal sequence of ICEPs as calculated by CFPF for the four frequency bands is shown. Below the results of the spatial correlation of the subsequent ICEPs are depicted, showing no stable temporal signature.

The datasets were split into 15 sections of equal length, each still longer than 2 min. For every section the EEG signal was split into 2 s segments corresponding to the TR used in the MR-acquisition. Within each section, for every segment, the mean frequency power over all channels for four frequency bands, i.e., delta: (2–4) Hz, theta: (4–8) Hz, alpha: (8–12) Hz, and beta: (12–30) Hz, was calculated, using a fast Fourier transformation (FFT), resulting in one time series for each frequency band. Motion related artifacts in the frequency power time courses were corrected. The frequency power time series were convolved with the standard SPM5 hemodynamic response function (HRF) and correlated with the RSN time courses taking into account common variance (partial correlation) between frequency bands. The correlation values were *Z*-transformed, using the mean over all correlation values across subjects as global mean, which resulted in time series of 15 *Z*-scores for every frequency band (see Figure 3). The temporal variance for each RSN and frequency band over the 15 time points was calculated and averaged over subjects (see Table 1). To estimate the temporal stability of ECPs within and across subjects, for each RSN and frequency band the *Z*-scores of the 15 sections were ranked from high to low, and averaged over subjects to visualize inter-subject variance.

**Table 1:** **Group mean temporal variance values (variance across the 15 sections) for GFPC and CFPF, as well as the mean temporal variance values of the same five subjects analyzed with SFPC and GFPC, for each RSN and frequency band**.

Freq band	RSN1	RSN2	RSN3	RSN4	RSN5	RSN6	RSN6b	RSN7	RSN8	RSN9	RSN10	RSN11	Average
** GFPC MEAN VARIANCE ACROSS ALL SUBJECTS**													
delta	1.029	1.080	0.985	1.036	0.857	1.081	1.050	0.805	1.031	0.882	1.091	0.936	**0.989**
theta	0.953	0.913	0.906	0.988	0.871	0.973	0.983	0.825	1.031	0.791	0.877	0.828	**0.912**
alpha	1.186	1.124	1.171	1.121	0.980	1.063	1.148	1.248	1.031	0.995	1.116	1.172	**1.113**
beta	1.193	1.219	0.981	0.961	0.855	1.060	1.124	0.994	1.031	0.943	1.102	1.136	**1.050**
**Average**	**1.090**	**1.084**	**1.011**	**1.026**	**0.891**	**1.044**	**1.076**	**0.968**	**1.031**	**0.903**	**1.046**	**1.018**	
** CFPF MEAN VARIANCE ACROSS ALL SUBJECTS**													
delta	0.620	0.501	0.695	0.566	0.667	0.658	0.590	0.731	0.685	0.650	0.635	0.669	**0.639**
theta	0.690	0.751	0.737	0.799	0.660	0.643	0.749	0.717	0.685	0.809	0.693	0.666	**0.717**
alpha	0.897	0.876	0.833	0.819	0.780	0.702	0.702	0.864	0.685	0.698	0.756	0.677	**0.774**
beta	0.798	0.842	0.856	0.759	0.767	0.763	0.808	0.827	0.685	0.743	0.839	0.755	**0.787**
**Average**	**0.751**	**0.742**	**0.780**	**0.736**	**0.719**	**0.691**	**0.712**	**0.785**	**0.685**	**0.725**	**0.731**	**0.692**	
** SFPC VARIANCE FOR FIVE SUBJECTS**													
delta	1.077	0.763	1.306	0.884	1.327	1.230	1.140	1.097	0.708	1.067	1.125	0.698	**1.035**
theta	0.587	0.829	1.004	0.791	1.241	1.330	1.018	0.840	0.708	0.889	0.882	0.965	**0.924**
alpha	1.658	1.582	1.366	1.550	0.671	1.508	1.511	1.763	0.708	1.081	1.491	1.095	**1.332**
beta	1.534	1.994	1.933	1.909	0.678	1.331	0.919	1.759	0.708	2.228	2.225	1.493	**1.559**
										**Mean variance five subjects**			**0.902**
** GFPC VARIANCE FOR FIVE SUBJECTS**													
delta	1.152	1.124	1.219	1.189	0.972	1.342	1.513	0.849	1.161	0.917	1.242	0.959	**0.987**
theta	0.812	0.944	0.923	1.121	0.923	1.190	1.135	0.867	1.161	0.734	0.949	0.860	**0.838**
alpha	1.448	1.315	1.325	1.253	0.885	1.323	1.503	1.317	1.161	0.990	1.336	1.109	**1.056**
beta	1.209	1.168	1.091	1.137	0.843	1.071	1.049	1.109	1.161	1.064	1.483	1.149	**0.994**
										**Mean variance five subjects**			**0.968**

*The variance values of GFPC and SFPC are comparable since both show the temporal variance of ECPs which represents the direct correlation between frequency power and RSNs. For CFPF the variance values represent the variance of the subsequent spatial correlation of the ICEPs, which is an indirect measure and not directly comparable to the other methods. However the overall huge temporal variance across all methods depicts the temporal instable relation between both modalities*.

### Source frequency power correlation

In order to get an indication for the effect of volume conduction and obtain more specific correlation patterns, in five subjects an in-house developed fMRI-informed source model was applied. In combination with a four layer realistic head model it enables to separate the EEG according to the fMRI-RSNs. This new method was tested in a separate study that employs a simple visual stimulation and is further referred to as Integrative Head Model (IHM). It merges FSL analysis, Freesurfer mesh generation (Freesurfer image analysis suite^2^), and a Neuroelectromagnetic Forward Head Modeling Toolbox (NFT) based head model (VER 2.0^3^; Acar and Makeig, 2010), to combine fMRI and EEG in an integrative way (see Figure 1). Tissue surface meshes (TSMs) from the individual T1 images are derived using Freesurfer and NFT. The scalp, inner and outer skull as well as brain TSMs are used in the Boundary Element Method (BEM) based forward model as implemented in NFT (Brain/scalp conductivity = 0.33 S/m, Skull conductivity = 0.0132 S/m, CSF conductivity = 1.79 S/m). The source space is constructed by seeding the cortical sheet with dipoles, the location, and orientation of which is derived from the pial and white matter TSMs. Sources are defined by selecting dipoles according to fMRI RSNs, mapped to the cortical sheet. A source is defined as the weighted vector sum of its active dipoles, where the weights are equal to the *Z* values of the fMRI activation map at dipole location, and normalized subsequently so that the sum of all weights within one source equals one. These fMRI derived sources are fed into the forward model (in NFT) to calculate the specific lead field matrix (LFM) using an electrode template, which was manually transformed to each subjects head. This specific LFM has a low dimensionality given by the number of sources times number of channel. It is inverted using a Moore–Penrose pseudo inverse and the inverted LFM is used to transform the EEG data to source specific time courses. This results in an EEG time course for each RSN. Furthermore, the same analysis steps as described in Method 1 were applied to the transformed EEG signal. For each of the 15 sections and each frequency band the fMRI derived source frequency power time courses were convolved with the standard SPM5 HRF, partial correlated with their associated RSN time course, and the correlation values were *Z*-transformed. The variance over the 15 sections was calculated and the *Z*-scores of the sections were ranked from high to low, to obtain an estimate of the temporal stability.

### Channel wise frequency power fit

After pre-processing, each channel of the EEG data was band pass filtered in four frequency bands [delta: (2–4) Hz, theta: (4–8) Hz, alpha: (8–12) Hz, and beta: (12–30) Hz] using an FFT-filter (EEGlab). Power time courses were obtained from the filtered data by applying a Hilbert transform and taking the squared magnitude of the resulting signal. To correct for movement, time points where the power estimate exceeded a threshold (seven times the mean of the time course) were set to the average of the time points immediately before and after. Power time courses were segmented in to 2 s segments, according to the TR used in the fMRI acquisition; subsequently each segment was averaged over time and the resulting frequency power time course for each channel were convolved with an HRF (SPM 5). Finally the HRF convolved frequency power time course and the RSN time courses were normalized to have zero mean and a standard deviation of one. Time courses of all ICs (including noise related components) were fitted to each frequency power time course in a separate GLM for every channel. This resulted in an estimate of signal contribution for each RSN to each electrode and EEG frequency band. Plotting these contribution estimates on a scalp plot, here termed independent component expression pattern (ICEP), gives a visual representation of the electrophysiological expression of the RSN for each frequency band. Applying this approach to each of the 15 sections resulted in 15 subsequent ICEPs representing their evolution over time.

In order to obtain a comparable estimate for this method, which gives a spatial distribution as opposed to a point estimate of the other two methods, the temporal stability of the ICEPs was assessed by calculating the spatial correlation between subsequent sections within one subject, RSN and EEG frequency band. For each of those, the correlation values were *Z*-transformed using bootstrap statistics and the *Z*-scores were averaged to obtain the mean over all combinations of sections. For the bootstrapped *Z*-transformation a distribution was generated by repeatedly (*n* = 10,000) selecting 15 ICEPs at random from the entire set of ICEPs for that subject applying the same spatial correlation analysis. For each RSN and frequency band the *Z*-scores of the 15 sections were ranked from high to low, and for group analysis averaged over subjects. Additionally the variance over the 15 sections as well as the average variance over subjects for each RSN and frequency band was calculated.

## Results

**Figure 4:**
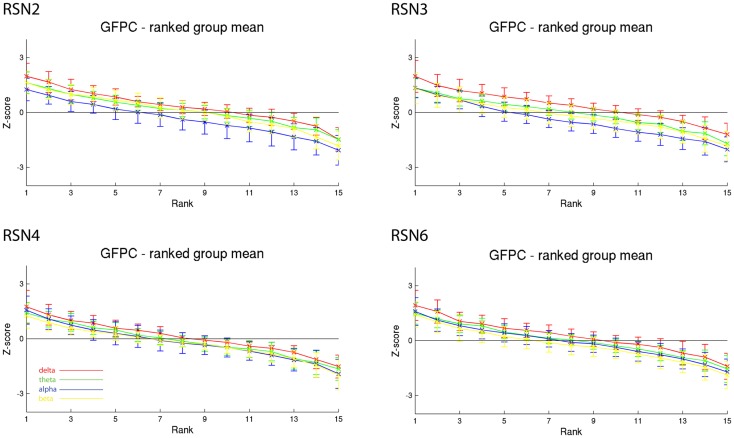
**Exemplary group results of RSN2 (occipital visual component), RSN3 (lateral visual component), RSN4 (DMN), and RSN6 (sensory motor component) for GFPC as rank graph showing large temporal variance within a subject**. The error bars (standard deviation across subjects) show the considerable inter-subject variability. Clearly alpha power shows a more negative correlation with the BOLD signal whereas delta power shows a more positive correlation for the visual components. Note that the connecting lines are only for visualization purposes.

**Figure 5:**
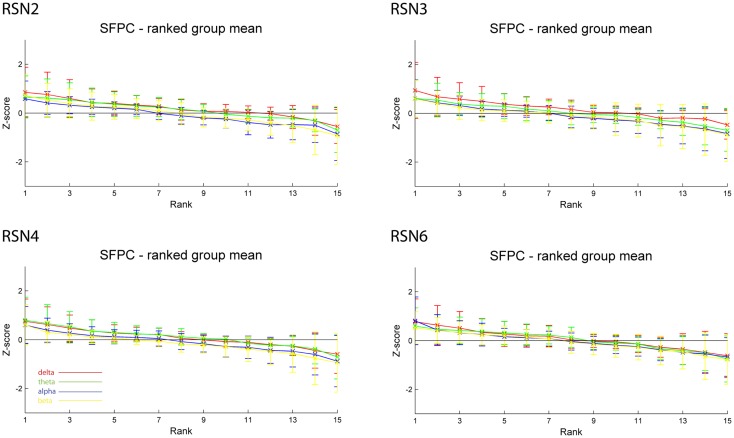
**Exemplary results of RSN2 (occipital visual component), RSN3 (lateral visual component), RSN4 (DMN), and RSN6 (sensory motor component) for the five analyzed subjects using SFPC comparable to Figure 4**. The error bars (standard deviation across subjects) show the considerable inter-subject variability, which is higher compared to GFPC; note that the *Z*-scores are smaller compared to GFPC. Despite these differences, also when using SFPC, alpha power shows a more negative correlation with the BOLD signal whereas delta power shows a more positive correlation for the visual components. The connecting lines are only for visualization purposes.

As reported in Meyer et al. (2013) we found reproducible fMRI RSNs across subjects (see Figure 2 for a depiction of the RSNs). In this study we observed very large inter-subject and intra-subject variability in the EEG frequency power correlations across all applied analysis methods. Figure 3 depicts the output of the different methods for one network (RSN3) of subject 1. It is clearly visible that GFPC and SFPC are not stable in time regarding their EEG frequency power correlation with the RSN time courses for all frequency bands. Figure 4 shows the results of the group analysis for GFPC and Figure 5 the results for the five subjects analyzed with SFPC. In both figures the group rank plots for four different RSNs show a large temporal variance within a subject – as reflected in the variance of the ranked *Z*-scores – in the depicted RSNs for all frequency bands. The error bars, indicating the standard deviation across subjects, show the considerable inter-subject variability. The error bars in Figure 5 are larger compared to those in Figure 4 which cannot be explained by the smaller number of analyzed subjects as controlled by performing GFPC on the same five subjects as for SFPC. Also note that, using SFPC the overall observed *Z*-scores are lower compared to GFPC. Strikingly, one can see in the ranking plots that for the visual components (see RSN2 and RSN3 in Figures 4 and 5) alpha power shows a more negative correlation with the BOLD signal whereas delta power shows a more positive correlation. This is also the case for the third visual component (not shown).

**Figure 6:**
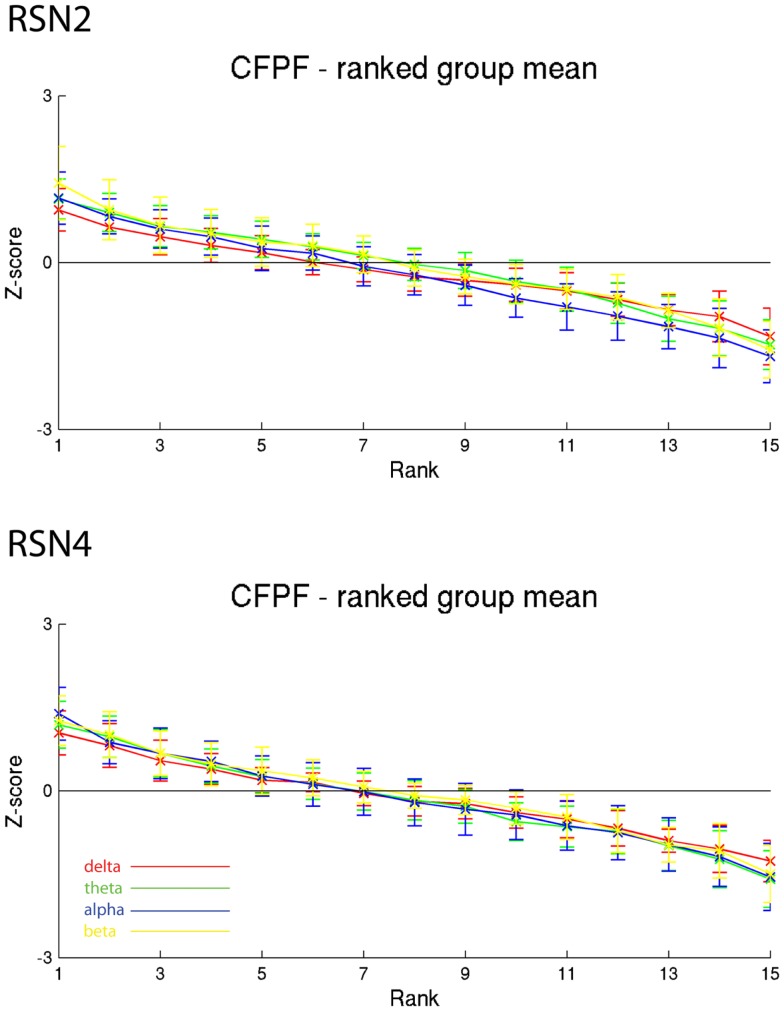
**Exemplary group results of RSN2 (occipital visual component) and RSN4 (DMN) for CFPF as rank graph showing no stable temporal signature**. The connecting lines are only for visualization purposes.

On the right hand side of Figure 3 the temporal sequence of ICEPs as calculated by CFPF for the four frequency bands as well as the results of the spatial correlation of the subsequent ICEPs is depicted. Clearly there is no temporally stable signature in the scalp maps for different sections of the dataset. This can be also observed in the group rank plot in Figure 6. The data shown in Figure 3 as well as the rank plots in Figures 4, 5, and 6 are typical examples for all analyzed subjects, all RSNs, and the four frequency bands examined, respectively. Table 1 summarizes the results containing the group mean temporal variance across the 15 sections, for each RSN and frequency band for GFPC and CFPF, respectively, as well as for the same five subjects analyzed with SFPC and GFPC.

## Discussion

The three methods used in this study were chosen to examine the temporal variability of ECPs from different perspectives with the aim to minimize methodological bias. GFPC is a very conservative approach with fairly little assumptions, taking the global EEG frequency power as independent parameter. However, due to the mixed nature of the EEG signal, volume conduction cannot be excluded, which might cause several sources contributing to a certain correlation and might explain temporally unstable ECPs. SFPC addresses this shortcoming by separating the EEG signal according to the fMRI-RSNs to correlate with, but this also did not result in temporally stable ECPs.

To test for possible methodological bias of GFPC and SFPC as source for the observed variance, we analyzed the data sets using a third approach. CFPF uses the RSN timelines as independent parameters and only uses the HRF to model the relation between the two modalities. Also this approach did not result in temporally stable correlation patterns. While the human HRF itself shows quite complex spatial dependencies (de Munck et al., 2007, 2009), in our correlation analysis it mainly causes a constant time shift and temporal smoothing, therefore it cannot be the reason for temporally instable correlation. Together with the findings of GFPC and SFPC, this leads to the suggestion that the analyzed low dimensional RSNs do not have a temporally stable relationship with EEG frequency band power fluctuations. However, the observed negative correlation of alpha power with the BOLD time courses for the visual components reaches statistical significance within three subjects for GFPC in agreement with previous literature (Goldman et al., 2002; Laufs et al., 2003a, 2006; Goncalves et al., 2006, 2008; Meyer et al., 2013), but does not reach statistical significance for either method on the group level.

One possible explanation for our observation of temporally instable ECPs might be given by Smith et al. (2012), who applied temporal ICA on high dimensional (200 spatial IC components) fMRI-RSNs and reported vast temporal dynamics within the lower dimensional (20–30 spatial IC components) RSNs. As such, there still might be a direct relation between RSNs and EEG frequency band power, but on a smaller spatial scale. However, one would expect a certain temporal stability in the results of SFPC and CFPF even if just a subcomponent of the low dimensional RSN expresses itself in a given EEG frequency band, which was not observed in our study.

An alternative explanation of our results would be, that during RS, frequency-specific power in the lower frequency bands of the EEG is not linked to changes in neuronal activity, reflected in changed oxygen consumption as measured by BOLD fMRI. This would also be supported by recent animal studies, e.g., Schölvinck et al. (2010), that show no stable correlation for the lower frequency bands in EEG with BOLD fMRI particularly in eyes open RS.

The observation that using SFPC reduced the overall observed *Z*-scores compared to GFPC gives rise to the assumption that the studied RSN characteristics are not related. However, one has to consider the potential limitation of the head model as used in our study; (a) the spatial resolution of the head model is limited by the relatively low number of electrodes and (b) its reduced spatial specificity in the context of spatially extended sources like RSNs, as we assume a concurrent temporal behavior within the whole source.

We therefore conclude that the correlation between lower frequency band power in EEG and BOLD RSNs time courses is at least temporally instable or even absent in eyes open RS.

## Conflict of Interest Statement

The authors declare that the research was conducted in the absence of any commercial or financial relationships that could be construed as a potential conflict of interest.

## Footnotes

^1^www.fmrib.ox.ac.uk/fsl

^2^http://surfer.nmr.mgh.harvard.edu/

^3^http://sccn.ucsd.edu/nft/
