# Corrigendum: The quest for EEG power band correlation with ICA derived fMRI resting state networks

**DOI:** 10.3389/fnhum.2014.00539

**Published:** 2014-09-02

**Authors:** Matthias C. Meyer, Ronald J. Janssen, Erik S. B. Van Oort, Christian F. Beckmann, Markus Barth

**Affiliations:** ^1^Donders Institute for Brain, Cognition and Behaviour, Radboud University NijmegenNijmegen, Netherlands; ^2^MIRA Institute for Biomedical Technology and Technical Medicine, University of TwenteTwente, Netherlands; ^3^Erwin L. Hahn Institute for Magnetic Resonance Imaging, University Duisburg-EssenEssen, Germany

**Keywords:** erratum, combined EEG-fMRI, resting state, source modeling, ICA, ECP

We have noticed that during the revision process of the original manuscript a modification in the analysis script to enable the parallel processing of more data sets led to incorrect indices for the selection of active dipoles. This mistake in the analysis pipeline affected the results of SFPC, i.e., Figure 5 and the part of Table 1 labeled “SFPC variance for 5 subjects.”

**Table 1:** **Correction of Table 1 in the original manuscript for GFPC and SFPC**.

	**RSN1**	**RSN2**	**RSN3**	**RSN4**	**RSN5**	**RSN6**	**RSN6b**	**RSN7**	**RSN8**	**RSN9**	**RSN10**	**RSN11**	**Average**
**GFPC 5 SUBJECTS**													
Delta	1.152	1.124	1.219	1.189	0.972	1.342	1.513	0.849	1.161	0.917	1.242	0.959	1.137
Theta	0.812	0.944	0.923	1.121	0.923	1.190	1.135	0.867	1.161	0.734	0.949	0.860	0.968
Alpha	1.448	1.315	1.325	1.253	0.885	1.323	1.503	1.317	1.161	0.990	1.336	1.109	1.247
Beta	1.209	1.168	1.091	1.137	0.843	1.071	1.049	1.109	1.161	1.064	1.483	1.149	1.128
													1.120													
**SFPC 5 SUBJECTS**													
Delta	1.000	0.931	0.863	0.861	1.185	0.938	1.248	1.000	0.891	1.137	0.786	0.913	0.979
Theta	1.015	0.859	0.843	0.951	0.856	0.920	0.906	0.843	0.891	1.160	0.979	0.980	0.934
Alpha	1.175	0.937	0.939	0.899	0.871	0.903	0.944	0.929	0.891	0.785	1.152	1.011	0.953
Beta	1.101	1.220	1.003	0.996	1.319	0.844	0.952	0.803	0.891	1.044	1.373	0.997	1.045
													0.978												
**SFPC CORRECTED 5 SUBJECTS**													
Delta	1.285	1.352	1.022	1.334	0.931	1.307	0.839	0.956	1.353	1.038	1.211	0.861	1.124
Theta	0.919	1.158	1.278	0.995	0.861	1.090	1.037	0.937	1.353	0.837	0.929	0.800	1.016
Alpha	0.764	0.844	0.812	1.012	0.717	0.946	0.891	0.980	1.353	0.757	0.913	0.873	0.905
Beta	0.948	1.197	1.069	0.855	0.927	1.075	0.901	1.021	1.353	0.816	1.185	0.799	1.012
													1.014												

*The values of GFPC and SFPC are the correction of the data transfer error. “SFPC Corrected” shows the new results for SFPC after re analysis of the 5 Subjects with the corrected analysis pipeline*.

We corrected this mistake in the analysis script and reanalyzed the 5 Subjects. While this affected the individual frequency power time courses, it did not result in a more stable correlation with the RSN timelines. The corrected Figure 5 of this erratum depicts the corrected rank graphs for SFPC, which show only minor differences to the erroneous graphs in the original Figure 5 of the published manuscript. This reflects a similar inter subject and temporal variance independent of the change in dipole location.

**Figure 1:**
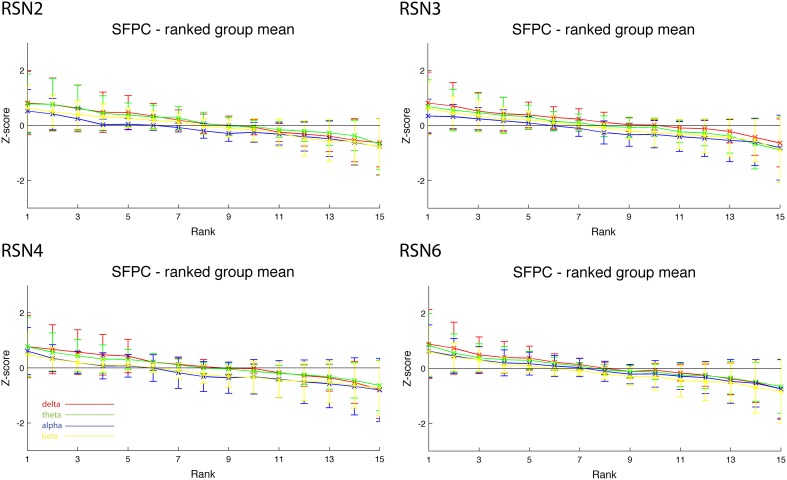
**Correction of Figure 5 of the original manuscript, showing only minor differences to the erroneous graphs in the original Figure 5**. This reflects a similar inter subject and temporal variance independent of the change in dipole location.

We also noted a lapse in the part of the original Table 1, which shows the variance values for SFPC and GFPC for 5 subjects. This was due to an error in the data transfer between Excel and Word in the final version of the manuscript after the revision process. The corrected Table 1 below shows the corrected values of both GFPC and SFPC analysis.

It is important to note that the corrected results did not impact on our original conclusions of the published manuscript.

## Conflict of interest statement

The authors declare that the research was conducted in the absence of any commercial or financial relationships that could be construed as a potential conflict of interest.

